# Retrospective Study on Mortality and Adrenal Insufficiency Following Emergency Intubation With Etomidate Versus Ketamine in Children

**DOI:** 10.7759/cureus.79581

**Published:** 2025-02-24

**Authors:** Donna Mendez, Krishna K Paul, Danielle O'Connell, Obadiah Michler, Heidi Schultz, Kelcie Hill, Dietrich V Jehle

**Affiliations:** 1 Department of Emergency Medicine, The University of Texas Medical Branch, Galveston, USA

**Keywords:** adrenal insufficiency, etomidate, ketamine infusion, pediatric, rapid sequence intubation (rsi)

## Abstract

Background: Etomidate is a sedative-hypnotic used for intubation in children. Its use in airway management has been controversial since etomidate may induce adrenal insufficiency. Ketamine is frequently used for intubation in children and has not been reported to be associated with adrenal insufficiency. We evaluated the risk of death and adrenal suppression after rapid sequence intubation (RSI) with either etomidate or ketamine.

Methods: This retrospective study was performed using the TriNetX database in the United States from 61 healthcare organizations (HCOs). The final cohort after propensity matching included 1,191 patients who were ≤17 years of age and were given etomidate or ketamine for RSI but not both. The time frame was from December 22, 2003, to October 22, 2022.

Results: After propensity matching, the etomidate and ketamine groups each contained 565 patients, and there was a significantly lower risk of adrenal suppression with etomidate (1.8%) compared to ketamine (4.2%) (RR=0.43, 95% CI=0.21-0.90, p=0.02). There were similar results regarding adrenal insufficiency when no propensity matching was performed. There were no differences in mortality when comparing intubation with etomidate or ketamine with and without propensity matching.

Conclusion: There was not an increased risk of adrenal insufficiency for etomidate administered as a single dose in children undergoing RSI in the emergency department (ED) when compared with ketamine. Additionally, there was no difference in mortality for those intubated with ketamine or etomidate.

## Introduction

Etomidate, an intravenous sedative-hypnotic agent, is widely used as a single dose for rapid sequence intubation (RSI) in both children and adults in emergency settings [1]. Its mechanism of action involves enhancing gamma-aminobutyric acid (GABA) activity at the GABA-A receptor, leading to increased chloride ion influx, hyperpolarization of neuronal membranes, and central nervous system depression. This results in sedation and hypnosis without significant analgesic effects. Etomidate’s rapid onset (30-60 seconds), short duration (5-10 minutes), and minimal hemodynamic effect make it particularly helpful for patients with unstable cardiovascular status, though adjunctive analgesics may be required [1].

Despite its clinical efficacy, etomidate has been associated with adrenal insufficiency, particularly in adult populations with long-term use but there is not much evidence that a single injection can cause adrenal insufficiency. In the 1980s, etomidate administered by continuous infusion was found to be excessively associated with mortality due to adrenal insufficiency, leading to the discontinuation of this practice [2,3]. Even as a single dose, etomidate has been suggested to interfere with adrenal steroidogenic enzymes, raising concerns about its safety profile [4,5]. While some of the adult studies where etomidate was given as a single injection suggest an association between etomidate and adrenal insufficiency [6,7], the evidence regarding its impact on mortality remains inconclusive [7-12].

In pediatric populations, the literature on etomidate’s effects on adrenal suppression and mortality is limited and conflicting. For instance, a retrospective study by Den Brinker et al. reported dose-dependent adrenal insufficiency for at least 24 hours following a single dose of etomidate in children with meningococcal sepsis, potentially increasing mortality risk [13]. Conversely, two separate studies involving approximately 100 pediatric patients each found no significant association between etomidate and adrenal suppression [8,14]. These discrepancies highlight the need for further investigation into the safety of etomidate in pediatric RSI.

Ketamine, another commonly used sedative agent for RSI, operates through a distinct mechanism of action as an N-methyl-D-aspartate (NMDA) receptor antagonist [15]. By blocking excitatory neurotransmission, ketamine produces a dissociative state characterized by analgesia, amnesia, and sedation. Unlike etomidate, ketamine provides both sedation and analgesia, preserves airway reflexes, and maintains respiratory drive at lower doses. Additionally, ketamine has not been associated with adrenal insufficiency, further distinguishing it from etomidate in terms of safety [15].

Given the ongoing controversy surrounding the association between etomidate and adrenal insufficiency, as well as the limited data on its use in pediatric patients, this study aims to evaluate the risk of death and adrenal suppression following emergency department (ED) intubation with etomidate versus ketamine in pediatric populations. By addressing this critical gap in the literature, the study seeks to provide information to help with decision-making in the emergency management of pediatric patients requiring RSI. This abstract was presented at the 2024 Society of Academic Medicine May 15, 2024, National Meeting.

## Materials and methods

Study design

This propensity-matched, retrospective study utilizing the TriNetX database was performed using information extracted from approximately 105 million patients from 61 healthcare organizations (HCOs) within the United States between December 22, 2003, and October 22, 2022. TriNetX is a database containing deidentified electronic medical records from the global federated health research network. The database includes demographic information as well as diagnoses, procedures, and medications administered. For this study, diagnoses were identified using the 10th revision of the International Statistical Classification of Diseases and Related Health Problems (ICD-10-CM) codes. Intubation was identified as a procedure using Unified Medical Language System Current Procedural Technology (UMLS:CPT:31500) codes. The clinical drugs used for intubation were identified using the U.S. National Library of Medicine RxNorm nomenclature NLM:RXNORM:4177 and NLM:RXNORM:6130 for etomidate and ketamine and NLM:RXNORM:10154 and NLM RXNORM:68139 for succinylcholine and rocuronium, respectively. TriNetX provides data that has been de-identified and as a result, IRB review was not required for this study.

The inclusion criteria were ≤17 years, intubated on the day of the ED visit, RSI in the ED with etomidate or ketamine, and received paralytics: succinylcholine or rocuronium. The exclusion criteria were a history of adrenal insufficiency or use of both etomidate and ketamine.

Outcomes

The outcomes evaluated were mortality and adrenal insufficiency within 60 days after the intubation. Patients were excluded from the analysis if they had a prior history of adrenal insufficiency, which was identified with ICD 10 codes (E27.1-4). Mortality data was obtained from EMR coding and HCOs, in combination with the national death registries. There is potential for missed death events when a patient is treated at an HCO not affiliated with the TriNetX network and subsequently experiences a fatal outcome outside of this network. However, this represents only a minor issue, as currently, 94% of HCOs within the TriNetX network are also linked to the United States death registries. This percentage is steadily increasing as more HCOs continue to be linked with the registries.

Analysis

This analysis compared the outcomes of two cohorts: group 1, children who received etomidate for RSI in the ED, and group 2, children who received ketamine for RSI in the ED. The measure of association tool in TriNetX was used to perform univariate analysis where risk ratios (RR), 95% CI, and probability values (P) were calculated to compare outcomes. Statistical significance was set at a two-sided alpha p<0.05. For the demographic data, chi-square was used for categorical variables such as race and gender. A t-test was used to analyze continuous variables. Propensity matching was performed for demographics and seven pre-existing conditions associated with mortality. Demographic variables included age, race, ethnicity, and gender. The preexisting conditions that were propensity-matched included acute and chronic kidney failure, obesity, cardiac disease, neurological diseases, severe sepsis, trauma, and chronic lower respiratory diseases.

## Results

There were 1,871 children intubated by RSI in the ED with etomidate (n=1,228) or ketamine (n=643) identified before propensity matching. After propensity matching, the etomidate and ketamine group each contained 565 patients, making the total cohort of 1,130 patients. Concerning the demographics before propensity matching, there were no differences in gender between the etomidate and ketamine groups. There was a difference in the age for the etomidate (9.4 years +/- 6.3 SD) versus the age for ketamine (6.4 years +/- 6.3 SD). When both groups were compared, there were more Hispanics or Latinos and Other races in the etomidate group versus the ketamine group. There were also significant differences in some pre-existing conditions. After propensity, there were no statistical differences in demographics or pre-existing conditions (Table 1).

**Table 1:** Characteristics of Pediatric Patients Given Etomidate (n=1,228) Versus Ketamine (n=643) for RSI *Cohort 1 = etomidate **Cohort 2 = ketamine RSI, rapid sequence intubation SD, standard deviance

		Before Propensity Score Matching					After Propensity Score Matching				
Demographics											
Cohort		Mean±SD	Patients	% of cohort	P-value	Standard difference	Mean±SD	Patients	% of cohort	P-value	Standard difference
1*	Age at index	9.4±6.3	1,228	100%	<0.001	0.474	6.7±6.2	565	100%	0.626	0.029
2**		6.4±6.1	643	100%			6.9±6.2	565	100%		
1	White		653	53.2%	0.908	0.006		317	56.1%	0.370	0.053
2			344	53.5%				302	53.5%		
1	Female		489	39.9%	0.484	0.034		227	40.2%	0.586	0.032
2			267	41.5%				236	41.8%		
1	Unknown ethnicity		70	5.7%	0.606	0.025		31	5.5%	0.791	0.016
2			33	5.1%				29	5.1%		
1	Not Hispanic or Latino		904	73.7%	<0.001	0.180		469	83.0%	0.281	0.064
2			522	81.2%				455	80.5%		
1	Hispanic or Latino		253	20.6%	<0.001	0.185		65	11.5%	0.156	0.085
2			88	13.7%				81	14.3%		
1	Black or African American		280	22.8%	0.395	0.041		137	24.2%	0.730	0.021
2			158	24.6%				142	25.1%		
1	Male		728	59.3%	0.673	0.021		336	59.5%	0.629	0.029
2			375	58.3%				328	58.1%		
1	Other Race		140	11.4%	0.001	0.171		40	7.1%	0.908	0.007
2			42	6.5%				41	7.3%		
1	Asian		43	3.5%	0.528	0.031		17	3.0%	1	<0.001
2			19	3.0%				17	3.0%		
Diagnosis											
Cohort		Mean±SD	Patients	% of cohort	P-value	Standard difference	Mean±SD	Patients	% of cohort	P-value	Standard difference
1	Acute kidney failure and chronic kidney disease		21	1.7%	0.001	0.147		19	3.4%	0.377	0.053
2			27	4.2%				14	2.5%		
1	Overweight and obesity		50	4.1%	0.774	0.014		18	3.2%	0.625	0.029
2			28	4.4%				21	3.7%		
1	Diseases of the circulatory system		111	9.0%	<0.001	0.277		80	14.2%	0.735	0.020
2			119	18.5%				84	14.9%		
1	Diseases of the nervous system		271	22.1%	0.003	0.144		148	26.2%	1	<0.001
2			182	28.3%				148	26.2%		
1	Other sepsis		24	2.0%	<0.001	0.251		21	3.7%	0.307	0.061
2			46	7.2%				28	5.0%		
1	Severe sepsis		10	0.8%	<0.001	0.218		10	1.8%	0.527	0.038
2			27	4.2%				13	2.3%		
1	Injury of unspecified body region		40	3.3%	0.720	0.018		12	2.1%	0.444	0.046
2			19	3.0%				16	2.8%		
1	Chronic lower respiratory diseases		111	9.0%	<0.001	0.220		79	14.0%	1	<0.001
2			105	16.3%				79	14.0%		

After propensity matching, there was a significant difference in the risk of adrenal suppression, with ketamine (4.2%) having a higher risk when compared to etomidate (1.8%) (RR=0.43, 95% CI=0.21-0.90, p=0.02). There were similar results when no propensity matching was performed (RR=0.20, 95% CI=0.10-0.20, p<.001). There were no differences in mortality when comparing intubation with etomidate or ketamine when propensity matching was performed (RR=0.95, 95% CI=0.67-1.35, p=0.78) (Table 2). When no propensity matching was performed, there also were no differences in mortality (RR=1.03, 95% CI=0.79-1.36,p=0.81) (Table 3). 

**Table 2:** Outcomes of Etomidate Versus Ketamine After Propensity Matching RR, relative risk *TriNetX conceals number ≤10 to protect patient privacy

Outcomes	Etomidate (%)	Ketamine (%)	RR (95% CI)	P-Value
Deceased	56 (10.0%)	59 (10.5%)	0.95 (0.67, 1.35)	0.78
Adrenal insufficiency	≤10* (1.8%)	23 (4.2%)	0.43 (0.21, 0.90)	0.20

**Table 3:** Outcomes of Etomidate Versus Ketamine Before Propensity Matching RR, relative risk *TriNetX conceals number ≤10 to protect patient privacy

Outcomes	Etomidate (%)	Ketamine (%)	RR (95% CI)	P-Value
Deceased	138 (11.0%)	69 (10.6%)	1.03 (0.79,1.36)	0.81
Adrenal insufficiency	≤10* (0.8%)	26 (4.1%)	0.20 (0.10,0.40)	<0.001

## Discussion

This study is the largest investigation comparing single-dose etomidate versus ketamine for RSI in children, focusing on mortality and adrenal insufficiency. The findings reveal no significant difference in mortality between ketamine and etomidate, regardless of whether propensity matching was applied. In contrast, the risk of adrenal insufficiency was minimally higher in the ketamine group after propensity matching, although this difference barely reached statistical significance.

Mortality associated with etomidate in the adult literature is inconsistent. Jabre et al., Punt et al., and Srivilaithon et al. found no increased mortality when comparing etomidate to ketamine [6-7,16]. Conversely, several other studies reported increased death with etomidate [9,16-18]. The 2015 Cochrane Review meta-analysis suggested a slight, though not statistically significant, increase in mortality with etomidate, but a higher incidence of positive adrenocorticotropic hormone stimulation (ACTH) tests, suggesting some degree of adrenal insufficiency [11].

Multiple adult studies have examined adrenal suppression related to etomidate and ketamine in RSI patients in the ED and intensive care unit (ICU). Jabre's 2009 randomized controlled trial of 655 patients found more of the etomidate patients had adrenal suppression between etomidate and ketamine, as assessed by ACTH stimulation tests and cortisol levels [6]. Smischeny et al. also found more adrenal suppression in those receiving a ketamine/propofol mixture versus etomidate in a randomized study [18]. A 2012 meta-analysis of seven studies found that etomidate increased the likelihood of adrenal suppression, though these studies did not compare etomidate to ketamine [11]. Conversely, Punt's 2014 study found no difference in cortisol levels between the two drugs, although both groups exhibited low cortisol levels 24 and 48 hours post-intubation [7].

In pediatric patients, only four studies have specifically examined adrenal suppression and mortality following etomidate administration. Den Brinker et al.'s 2008 study found higher mortality and adrenal suppression in the etomidate group compared to those receiving other sedatives like opioid agonists, propofol, ketamine, or midazolam [13]. Another study by Den Brinker on children with meningococcal sepsis also reported higher adrenal suppression with etomidate when compared to the same group of opioid agonists [19]. Sokolov et al.'s study of 100 children less than 10 years old found no adrenal suppression with etomidate, though the classification was based solely on the administration of corticosteroids at hospitalization, without direct measurement of cortisol levels or ACTH tests [8]. The most recent study, done by Guidner et al., found a low risk of adrenal insufficiency with etomidate when compared to lidocaine, morphine, and midazolam. Their definition of adrenal insufficiency included children who died or required administration of corticosteroids for confirmed or suspected adrenal insufficiency (14).

Etomidate is associated with adrenal suppression and mortality due to its inhibition of 11β-hydroxylase, which is essential for cortisol synthesis. Ketamine is rarely associated with adrenal suppression, and when it is, the suppression is usually attributed to the patient’s critical illness. In Punt's study, both ketamine and etomidate were linked to decreased cortisol levels, likely due to critical illness [7]. Jabre's study also found adrenal suppression in about half of the ketamine group, measured by ACTH stimulation tests, which was also speculated to be due to critical illness [6]. An animal study utilizing a sepsis model on mice showed that both etomidate and ketamine down-regulated genes involved in corticosteroid synthesis but it is not entirely clear how these findings apply to humans. In this study, ketamine was associated with a minimally higher risk of adrenal insufficiency compared to etomidate. Despite propensity matching for severe sepsis, ketamine may have been administered more often to critically ill patients due to its ability to increase blood pressure and modulate the systemic inflammatory response [20-25].

Critical illness has been reported to be independently associated with adrenal suppression, regardless of whether etomidate or ketamine is used. Malerva et al. found that about half of ICU patients on mechanical ventilation exhibited adrenal suppression, as measured by corticotropin tests [23]. Cooper et al. reported that 51% of septic shock patients who had not received etomidate had adrenal suppression, measured by ACTH stimulation tests [26]. Factors like low pH, bicarbonate levels, platelet count, disease severity, and organ failure are linked to adrenal suppression in critically ill patients. Critical illness is also associated with a decrease in cortisol binding protein, impairment of enzymes of the steroidogenic pathway before 21-hydroxylase, ACTH receptor insensitivity, decreased levels of cholesterol, decreased adrenal blood flow during severe shock, and adrenal hemorrhage [27-30].

The main limitations of this study include its retrospective design, which limits the ability to establish causality. There are difficulties with capturing clinical signs, vital signs, and laboratory measures from the TriNetX database, which may have led to inadequate propensity matching. Additionally, we relied on ICD-9 and ICD-10 codes for diagnosing adrenal insufficiency, which were assigned at the physician's discretion, making it unclear whether adrenal insufficiency diagnoses were based on ACTH stimulation tests, steroid administration, or cortisol levels. Ketamine is often used for intubating children with low blood pressure or those appearing clinically sicker because of its properties to increase blood pressure [20-24]; however, we performed propensity matching for sepsis in an attempt to control for this issue. However, clinical measures of severity of illness, such as clinical signs, vital signs, and laboratory measures, were not included. The study focuses on RSI, which generally utilizes a single dose of etomidate or ketamine. It is possible that some patients could have received multiple doses or additional medications that could influence outcomes. Finally, adrenal insufficiency and mortality were the primary outcomes, but the study did not evaluate other potential complications or long-term outcomes associated with the use of etomidate or ketamine in pediatric patients.

## Conclusions

In conclusion, our study attempted to address the issue of etomidate in pediatric RSI with a large database, an issue that has not been fully investigated. This could change current practice if the effects are fully understood. In our study, a single-dose etomidate for RSI in children in the ED does not appear to increase the risk of adrenal insufficiency compared to ketamine. No difference in mortality was observed between the two drugs. Future studies should include clinical signs, vital signs, and laboratory measures to better delineate adrenal insufficiency risk in children undergoing RSI with etomidate and ketamine.
